# America’s electorate is increasingly polarized along partisan lines about voting by mail during the COVID-19 crisis

**DOI:** 10.1073/pnas.2008023117

**Published:** 2020-09-22

**Authors:** Mackenzie Lockhart, Seth J. Hill, Jennifer Merolla, Mindy Romero, Thad Kousser

**Affiliations:** ^a^Department of Political Science, University of California San Diego, La Jolla, CA 92093;; ^b^Department of Political Science, University of California, Riverside, CA 92521;; ^c^Center for Inclusive Democracy, University of Southern California, Los Angeles, CA 90007

**Keywords:** elections, COVID-19, governance, partisanship

## Abstract

Are voters as polarized as political leaders when it comes to their preferences about how to cast their ballots in November 2020 and their policy positions on how elections should be run in light of the COVID-19 outbreak? Prior research has shown little party divide on voting by mail, with nearly equal percentages of voters in both parties choosing to vote this way where it is an option. Has a divide opened up this year in how voters aligned with the Democratic and Republican parties prefer to cast a ballot? We address these questions with two nationally diverse, online surveys fielded from April 8 to 10 and June 11 to 13, of 5,612 and 5,818 eligible voters, respectively, with an embedded experiment providing treated respondents with scientific projections about the COVID-19 outbreak. We find a nearly 10 percentage point difference between Democrats and Republicans in their preference for voting by mail in April, which had doubled in size to nearly 20 percentage points in June. This partisan gap is wider still for those exposed to scientific projections about the pandemic. We also find that support for national legislation requiring states to offer no-excuse absentee ballots has emerged as an increasingly polarized issue.

Facing the threat of the COVID-19 crisis, elected officials have offered divergent prescriptions about how to run November’s election. Democratic Senators Klobuchar and Wyden have introduced legislation to expand access to voting through the mail and some states have proposed to run their primaries entirely through the mail (1). Republican leaders have spoken against the mandatory mail ballot approach (2); President Trump has tweeted that “Republicans should fight very hard when it comes to state-wide mail-in voting. Democrats are clamoring for it. Tremendous potential for voter fraud, and for whatever reason, doesn’t work out well for Republicans” (3).

Among voters, prior research has shown little party divide on voting by mail, with nearly equal percentages of voters in both parties choosing to vote this way in past studies (4, 5). Has a divide emerged this year in how voters aligned with the Democratic and Republican parties want to cast a ballot?

To provide data that inform these conversations, we report how two samples of eligible voters want to see November’s election run. We present the results of two online surveys fielded on April 8 to 10 and then June 11 to 13 asking a nationally diverse sample of 5,612 and 5,818 eligible American voters for their views on how they would like to vote in November and their preferences about proposed changes in election policies. We embed a randomized experiment in these surveys presenting respondents with truthful summaries of the projections of two teams of scientists about the COVID-19 outbreak (6, 7): one predicting a peak in the spring and the other in the fall.

We preregistered three hypotheses. First, in line with previous research, we expected to observe no partisan gap in personal preference over how to cast a ballot for respondents in the control condition (H1). By contrast, we predicted that there would be partisan divides on support for national legislation expanding no-excuse absentee ballot availability (H2). Third, because recent work has shown that Republicans are less concerned with COVID-19 and less trusting of scientific efforts, the impact of expert COVID-19 projections in the treatment conditions should be attenuated for Republicans (H3). We find evidence against H1: A significant partisan divide has emerged in 2020 over how respondents prefer to cast their ballots. As expected, we also observe partisan polarization over vote-by-mail policies and differential treatment effects for respondents in different parties. We also show that the gaps in personal voting preferences and in views on election policy have grown significantly during the COVID-19 pandemic.

## Results

Table 1 displays results from two models testing for partisan differences. These results are from ordinary least squares (OLS) regressions where respondents from the nationally diverse samples are weighted to match the sample frame of the national citizen, voting-age population and the *P* values are from one-tailed hypothesis tests based on the preregistered hypotheses. We pool treatment conditions because the effects of the treatments were not substantively different; all treated respondents read one of two scientific projections about COVID-19.

**Table 1.** Results from weighted OLS regressions of support for no-excuse absentee balloting and vote-by-mail balloting on treatment, partisanship, and their interaction

	Dependent variable	
	Personal preference is to vote by mail (1)	Support for no-excuse absentee ballots (2)
Treatment	0.075**	−0.013
	(0.014)	(0.014)
Independent	−0.008	−0.287**
	(0.026)	(0.027)
Republican	−0.096**	−0.232**
	(0.020)	(0.019)
Treatment × Independent	0.007	0.046
	(0.028)	(0.029)
Treatment × Republican	−0.057**	0.040*
	(0.021)	(0.020)
June survey	0.047**	−0.023*
	(0.014)	(0.013)
June survey × Independent	−0.041	0.013
	(0.027)	(0.028)
June survey × Republican	−0.097**	−0.103**
	(0.020)	(0.019)
Constant	−0.041	0.873**
	(0.013)	(0.013)
Observations	11,223	9,393
R^2^	0.036	0.088
Adjusted R^2^	0.036	0.088

*P < 0.05; **P < 0.01. *P* values based on one-tailed tests.

Column (1) reports results from a model predicting a personal preference for voting by mail, rather than in person. In the control condition, Democrats were significantly more likely than Republicans to want to cast their own ballot by mail and this gap has grown since April. In April, 40.1% of Democrats indicated that they would like to vote by mail in November while 30.5% of Republicans wanted to vote by mail. In June, this gap doubled as 44.8% of Democrats and only 25.5% of Republicans indicated they would like to vote by mail at this point.

Column (2) shows support for national legislation requiring states to implement no-excuse absentee ballots for all voters who request them, among those offering an opinion. Support is relatively high; in April, 87.3% of Democrats supported the policy and 64.1% of Republicans supported it. The coefficient representing the change between surveys shows a small drop in support for no-excuse absentee ballots for Democrats (2.3%) but a much larger decline for Republicans (12.6%), suggesting an increasing partisan divide on the issue.

Turning to the experiment, the treatment moved Democratic respondents toward preferring to vote by mail in November. Reading scientific projections of when COVID-19 cases are likely to peak increased Democrats’ preference to vote by mail by 7.5 percentage points. Republicans showed a different pattern, as the treatment effect on the respondent’s preferred method to cast a ballot was significantly lower (and indistinguishable from 0). For policy preferences, Democrats were largely unmoved by the treatment, possibly due to very high levels of baseline support, while Republicans were moved slightly, shrinking the gap between partisans.

**Fig. 1.**
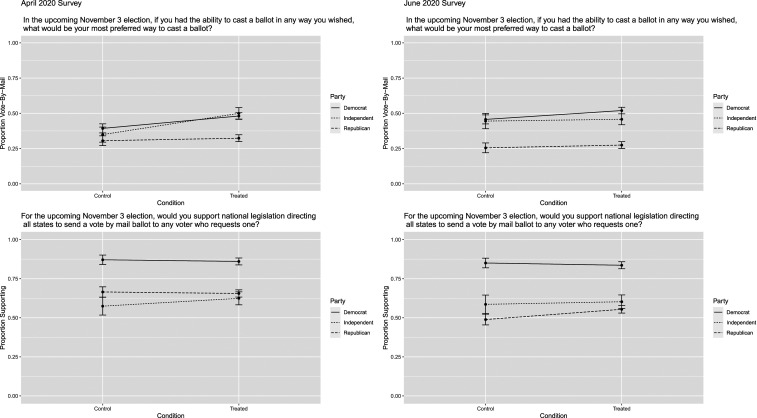
Predicted probabilities for supporting voting by mail or preferring to vote by mail by party and treatment condition in April (*Left*) and June (*Right*).

Fig. 1 provides predicted probability plots showing the likelihood of choosing voting by mail as one’s preferred method of voting and supporting no-excuse absentee ballot legislation across conditions and partisanship. This figure highlights that scientific projections related to COVID-19 widened the partisan divide in vote-by-mail preferences and also the growing polarization on vote-by-mail policies between April and June.

## Discussion

These results shed light on how COVID-19 and the current national political climate might alter previous findings on voting by mail. While previous work showed no partisan gap related to voting by mail in American elections (4), our results suggest that COVID-19, along with the statements by today’s party leaders, has the potential to create one.

Confirming our hypothesis, we found significant partisan differences in support for no-excuse absentee ballot legislation; a strong majority of Democrats support such a proposal while Republicans are more evenly split, although a majority also support it. Because this policy is but one of many possible approaches—9 states will automatically mail ballots for the 2020 Election and 10 will send out vote-by-mail applications, while 8 states require an excuse beyond COVID-19 fear to vote absentee (8)—an important avenue for further research would be to explore partisan divides over other election reforms in this realm.

Contrary to our expectations, we found a significant difference in partisan preference to personally vote by mail; nearly a third of Republicans do prefer this option for November’s election, but Democrats (as well as independents) are significantly more likely to want to cast their ballot without visiting a polling place.

We also found that personal preferences to vote by mail and support for national legislation expanding voting by mail have become increasingly polarized since April, with the gap in preferences over how to vote almost doubling. One potential explanation for this is increasing media coverage that frames voting by mail as a partisan issue.

The experimental results show that exposure to information about the peak of the COVID-19 outbreak exacerbates the partisan gap, with Democrats becoming significantly more likely to want to cast their ballot by mail while the same information did not move Republican preferences. This finding matches existing research on the partisan divide in trust in experts (9); Republicans are less willing to trust expert advice than Democrats. If the COVID-19 outbreak continues to worsen, this might contribute to a widening divide in partisan preferences on how to cast a ballot.

How will this affect the November election? This may depend upon local health conditions and a state’s election laws. If all voters have access to their preferred method of voting and feel safe casting a ballot through that method, Republicans could vote in person at high rates while many Democrats shift toward voting by mail, with no net impact. However, for Republicans in areas with high infection rates, concerns about COVID-19 exposure combined with reluctance to cast a mail ballot may lead to lower participation. A recent report shows that in Wisconsin’s 2020 presidential primary, in which 59% of votes were cast by mail, “Republican turnout declined near uniformly across the state relative to 2016” (10). For Democrats or independents who would now prefer to vote by mail, the laws in some states that limit vote-by-mail opportunities—such as those challenged by voting rights groups before Georgia’s 2020 primary (11)—could decrease their turnout. Whether the expressed preferences will result in actual partisan differences in voting behavior will be revealed in November 2020.

## Materials and Methods

The surveys were fielded from April 8 to 10 and June 11 to 13, 2020 using Luc.Id’s Fulcrum platform. This platform has been demonstrated to provide nationally diverse samples that exhibit treatment effects similar to samples from other sources (12). The sample frame was American citizens of voting age. The total sample included 5,612 and 5,818 respondents, respectively. Partisanship was measured using a 7-point scale that ranges from “Strongly Democrat” to “Strongly Republican” and both those who identified with or indicated they leaned toward one of the two major parties were included as partisans. The treatments were as follows:

• While no one can be certain how the COVID-19 outbreak will progress in the United States, one well-respected team of scientists at a leading university has projected that if social distancing measures are widely adopted [, the effects of the virus will [reach/have reached] their peak in April, then gradually decline throughout the spring and into the summer./now but are lifted during the early fall, a new surge in cases will come and the effects of the virus will reach their peak in November or December.]

This study was reviewed by the institutional review board at the University of California San Diego and deemed exempt as it met the criteria for minimal risk and participants were debriefed at the end. Informed consent was collected before respondents answered any questions. The method and hypotheses were preregistered at https://osf.io/ke8w2. Data and associated protocol will be available on Dataverse at https://dataverse.harvard.edu/dataset.xhtml?persistentId=doi:10.7910/DVN/GF0XJA (13) and are also described in the working paper posted at https://escholarship.org/uc/item/6mb7764n.

## Data Availability

Survey results data have been deposited in Dataverse (13).
